# Clinical Characteristics and Outcome of Patients With Pheochromocytoma: A Single Center Tertiary Care Experience

**DOI:** 10.7759/cureus.7990

**Published:** 2020-05-06

**Authors:** Sara Sohail, Waqas Shafiq, Syed Abbas Raza, Adnan Zahid, Khurram Mir, Umal Azmat

**Affiliations:** 1 Endocrinology and Diabetes, Shaukat Khanum Memorial Cancer Hospital and Research Centre, Lahore, PAK; 2 Surgical Oncology, Shaukat Khanum Memorial Cancer Hospital and Research Centre, Lahore, PAK

**Keywords:** adrenal medulla, pheochromocytoma, catecholamines, plasma metanephrine and normetanephrine levels, urinary vanillylmandelic acid (24-hour urinary vma)

## Abstract

Objective

The aim of this study was to evaluate the demographic and clinical characteristics of patients with pheochromocytoma and determine the treatment outcome with overall survival rates of patients with pheochromocytoma.

Methods

A retrospective, cross-sectional study was performed on all the patients with histologically proven pheochromocytoma presenting to Shaukat Khanum Memorial Cancer Hospital and Research Center (SKMCH & RC) Lahore, between August 2006 and July 2018. Clinical, biochemical and radiological data were collected at presentation, post-surgery, at discharge and till the last follow-up; data was retrieved from hospital records. Cure was defined as no evidence of disease biochemically, clinically, and structurally.

Results

This study included 29 patients, 69% were female. The three most common symptoms were abdominal pain (51.7%), hypertension (44.8%) and headache (41.4%). Most pheochromocytomas were sporadic (82.8%), all were adrenal gland tumors, and 89.7% were unilateral. All patients underwent adrenalectomy. Open adrenalectomy was performed in 25 patients whereas four underwent laparoscopic adrenalectomy. Fifteen patients experienced postoperative complications. The most frequently documented intraoperative complication was hypotension. Death occurred in two patients, one patient died of metastatic disease secondary to malignant pheochromocytoma and the other patient died from perioperative complications. Cure was documented biochemically and/or radiologically in 96.5% patients.

Conclusions

Abdominal pain was predominant presenting feature most likely due to large tumor size. Most patients presenting to SKMCH & RC, had large intra-abdominal tumors with high cure rate. Mortality was low despite high rate of perioperative complications.

## Introduction

Pheochromocytoma is a tumor arising from adrenomedullary chromaffin cells that commonly produces one or more catecholamines [1]. About 80% to 85% of all chromaffin-cell tumors are pheochromocytomas [2]. The incidence of pheochromocytoma is two to eight per million persons per year [3]. It is found in 0.1% to 1% of patients presenting with hypertension and as Young et al. reported approximately 5% of patients with incidentally discovered adrenal masses [4-6,7]. The peak incidence occurs in the third to fifth decade of life. The mean age at diagnosis is 24.9 years in hereditary cases and 43.9 years in sporadic cases [8]. It occurs more often in females than in males [9]. Most catecholamine secreting tumors are sporadic. However, about 25% of patients have the disease as part of a hereditary syndrome [8-10]. The inherited familial syndromes associated with pheochromocytoma include multiple endocrine syndrome type 2 (MEN 2), von Hippel-Lindau (VHL) syndrome and neurofibromatosis type 1 (NF-1) [2,11]. Pheochromocytoma has malignant potential as well. Malignancy is defined as the presence of metastases in non-chromaffin tissue. The prevalence of malignancy varies between 10% and 17% [12].

The classic triad of clinical features consists of episodic headaches, palpitations, and diaphoresis [13]. The hallmark clinical finding is hypertension, which can be paroxysmal or sustained. Hypertensive crisis can lead to cardiac arrhythmias, myocardial infarction, and even death. When the tumor is suspected clinically, biochemical confirmation, consisting of plasma and urinary catecholamine (adrenaline, noradrenaline) and their metabolites (metanephrine and normetanephrine) is required. This should be followed by tumor localisation using various imaging studies [2,11]. Computed tomography (CT) imaging or magnetic resonance imaging (MRI) of the abdomen and pelvis (at least through the level of the aortic bifurcation) are the most commonly used methods and have similar sensitivities (90%-100%) and specificities (70%-80%) for tumor localisation [14]. Surgical resection followed by postoperative monitoring is the safest and the most effective therapeutic intervention for pheochromocytoma [15].

Data regarding survival of patients with localized (benign) disease or regional disease is not clearly available. Patients with localized disease are expected to experience an overall survival approaching that of age matched disease-free individuals but 6.5% to 16.5% of these patients will develop a recurrence, usually 5 to 15 years after initial surgery and approximately 50% of the patients with recurrent disease experience distant metastasis [16,17]. The five-year survival in the setting of metastatic disease (whether identified at the time of initial diagnosis or identified post operatively as recurrent disease) is 40% to 45% [18]. Long-term follow-up is essential for all patients with pheochromocytoma even when initial pathology demonstrates no findings that are concerning for malignancy. After resection of a solitary sporadic pheochromocytoma, patients should undergo baseline postoperative biochemical testing followed by annual lifelong biochemical testing. Patients with hereditary syndromes of pheochromocytoma who have undergone resection, in addition to lifelong annual biochemical screening of pheochromocytoma, routine screening for other component tumors of their specific syndrome is indicated [6].

There are several reports and data available on pheochromocytoma from centres in developed countries, but there is limited information available from developing regions of the world [19,20]. This can be related to limited healthcare facilities, unavailability of specific tests for diagnosis of pheochromocytoma. Single institution experience is also limited in our part of world with paucity of local data. Due to rarity of this tumor, to date there is no study conducted on pheochromocytoma patients, on their various presentation and outcomes from Pakistan. The aims of this study were to look into the demographics, different clinical presentation of pheochromocytoma, to determine the treatment outcome of these patients. This study will further help in recognition of symptoms and early referral leading to proper management of this tumor.

## Materials and methods

A retrospective cross-sectional study was performed on all the patients with pheochromocytoma presenting to Shaukat Khanum Memorial Cancer Hospital and Research Centre (SKMCH & RC) Lahore, between August 2006 and July 2018. The study was approved by Institutional Review Board committee of SKMCH & RC (EXMPT-13-08-18-01).

Clinical information on all patients with histologically proven pheochromocytoma was retrieved from hospital records. For each patient, information at presentation, post-surgery, at discharge and till the last follow-up was recorded by the primary author and noted in a predefined questionnaire. No patients were excluded from the study over the study period.

Laboratory tests were done at SKMCH & RC. Pre- and post-surgery hormonal assessment for pheochromocytoma was done for all patients and was retrieved from hospital records and noted in questionnaire. Until 2015, 24-hour urine vanillylmandelic acid (VMA) test was performed. After 2015, plasma metanephrine and normetanephrines levels were standard hormone assessment for these patients.

Radiological investigations were performed which included computed tomography scan (CT scan), magnetic resonance imaging (MRI) and Iodine I 123-metaiodobenzylguanidine (MIBG) scintigraphy. Tumor size was recorded on histology samples and was retrieved from patient’s hospital record. Pheochromocytoma of adrenal gland scoring scale (PASS Score) based on different histological parameters, was calculated [21].

Postoperative patients were followed biochemically and radiologically at regular interval. Biochemical cure was defined as normal plasma metanephrine and normetanephrine levels post-operatively. Structural cure was determined by absence of tumor on radiological imaging post-surgery. For the outcomes, inpatient and outpatient notes, presurgery and post-surgery biochemical results, and scans of patients’ follow-up at hospital after their surgical procedures were being reviewed.

Furthermore, age, sex, body mass index, tumor characteristics (tumor stage, grade, histology) were treated as independent variables. Outcome response (alive or death) was treated as dependent variable.

Statistical Package for the Social Sciences (SPSS) version 20 (IBM Corp., Armonk, NY) was used for statistical analysis. Descriptive statistics were used and categorical data was analysed by calculating frequencies, percentages (gender). Continuous variables were reported in terms of mean and standard deviation (age).

## Results

A total of 29 patients (20 females) were included in the study. Mean age at presentation was 36.2 + 12.5 years. The majority of patients were from Punjab (55.2 %) followed by Khyber Pakhtunkha (KPK) (31.0%) and Afghanistan (13.8%). The rest of the demographic data and baseline characteristics are given in Table 1.

**Table 1:** Baseline characteristics of patients with pheochromocytoma at presentation (n = 29) BMI: Body Mass Index

Characteristic	N (%)
Age	
Mean	36.24 + 12.49 years
Range	20 to 64 years
Gender	
Male	9 (31%)
Female	20 (69%)
BMI (kg/m^2^)	
Mean	25.77 + 14.59
Range	18.29 to 31.64
Co-morbidity at Presentation	
Diabetes	5 (17.2%)
Hypertension	3 (10.3%)

The three most common clinical presentations were abdominal pain (51.7%), hypertension (44.8%) and headache (41.1%), whereas pheochromocytoma was an incidental finding in four patients (13.8%). The classical triad of headache, palpitation and sweating was seen in only three patients (10.3%). At presentation, mean heart rate was 87.7 beats per min (62-130), mean systolic blood pressure was 140.9 mm Hg (110-210) and mean diastolic blood pressure was 89.15 mm Hg (60-150). Two of our cases presented with hypertensive crisis, one with vision loss secondary to malignant hypertension and the other developed hypertensive crisis during and invasive procedure. The various different clinical features at presentation in patients with pheochromocytoma are detailed in Table 2.

**Table 2:** Clinical features at presentation in patients with pheochromocytoma (n = 29) PASS Score: Pheochromocytoma of adrenal gland scoring scale

Clinical Presentation	Total Percentage
Abdominal Pain	15 (51.7%)
Hypertension	13 (44.8%)
Headache	12 (41.1%)
Palpitation	10 (34.5%)
Sweating	5 (17.2%)
Abdominal Mass	5 (17.2%)
Incidental	4 (13.8%)
Classical Triad	3 (10.3%)
Tumor Laterality	
Right sided	14 (48.3%)
Left sided	12 (41.4%)
Bilateral	3 (10.3%)
Tumor Size (cm)	
Mean	8.31 + 3.5
Range	4 to 18 cm
PASS Score	
PASS Score < 4	20 (69%)
PASS score > 4	6 (20.7%)
PASS Score not available	3 (10.3%)

All patients presented with intra-abdominal tumors that originated from adrenal gland. Only three (10.3%) of these patients had bilateral pheochromocytoma.

The majority (82.8%) of patients had sporadic tumors. Five (17.2%) patients had familial syndromes MEN type 2a based on the presence of medullary thyroid cancer and pheochromocytoma concomitantly. Pheochromocytoma was bilateral in three (60%) and unilateral in two (40%) of these patients.

Plasma metanephrine and normetanephrine levels were checked in 19 patients (65.5%), 24-hour urinary VMA levels were checked in six patients (20.7%). Hormonal assessment was not available in four patients (13.8%). Twelve patients (63.2%) had increased plasma metanephrine and normetanephrine levels and seven patients (36.8%) had increased normetanephrine levels only. Twenty-four hour urinary VMA levels were normal in all six patients (20.7%).

At presentation, all patients had CT scan for initial tumor localisation. Eleven patients had MRI scans; only one patient showed evidence of metastasis. In 26 patients, CT or MRI scans correctly identified tumor site before surgery. In two patients, tumor was initially diagnosed as pancreatic tail mass and in one patient tumor was initially diagnosed as extra adrenal which later, on further imaging studies, revealed an adrenal mass. Baseline MIBG scans were done in 20 patients; of these all correctly identified the tumor preoperatively.

All patients underwent adrenalectomy. Open adrenalectomy was performed in 25 patients whereas four patients underwent laparoscopic adrenalectomy. Perioperative complications are presented in Table 3.

**Table 3:** Therapeutic modalities and perioperative complications in patients with pheochromocytoma (n = 27)

Complications	Preoperative Alpha and Beta blockade (n = 20)	No medical management preoperative (n = 7)
Perioperative Complications	10	5
Hypotension	9	2
Haemorrhage	1	1
Hypertensive crisis	none	2

Twenty patients received alpha and beta blockade prior to surgery. Postoperative complications were reported in 15 patients and they were more common in patients who did not receive preoperative alpha and beta blockade. There was no death reported in perioperative period.

On histopathology mean tumor size was 8.3 cm (5 cm to 18 cm). PASS Score was calculated in 26 patients. Twenty patients were noted to have PASS score less than 4 and the rest of six patients had PASS score greater than equal to 4. Immunohistochemical staining (IHC) was performed in 27 patients, details are listed in Table 4.

**Table 4:** Immunohistochemical staining pattern in pheochromocytoma patients

Immunohistochemical staining	Number of patients
Chromogranin	9
Synaptophysin	2
Synaptophysin and Chromogranin	6
Synaptophysin and S-100	1
Synaptophysin + Chromogranin + S-100	4
Chromogranin and S-100	5

Complete biochemical and structural cure post surgery was achieved in 28 patients. Normalization of blood pressure was achieved in all pheochromocytoma patients who presented with hypertension as initial clinical finding. Mean patient follow-up was 16.5 months (two months to 58 months). None of these patients showed evidence of disease recurrence except one patient who presented with metastatic disease. Two mortalities were recorded, one patient died due to metastatic pheochromocytoma, the second patient died of adrenal crisis as she had undergone bilateral adrenalectomy for bilateral pheochromocytoma.

## Discussion

Our study has shown that peak incidence of pheochromocytoma occurs in the fourth to fifth decades of life and is more common in female. There was no extra adrenal pheochromocytoma in our study. On average, every sixth patient had pheochromocytoma as a part of a hereditary syndrome (MEN-2A). These results were lower than that reported by Safwat et al. (n = 17, 14.9%) [22].

The most frequent clinical presentation was abdominal pain (likely secondary to mass effect) followed by hypertension. The classical triad (headache, palpitations, sweating) was only present in minority of patients. Similar presentations have also been reported in other studies as well [22,23]. These studies have reported a higher rate of hypertension than we observed. One possible reason for this is that about one-sixth of our patients had medullary thyroid cancer (MTC) and only on further investigations were found to have pheochromocytoma and did not initially present to us for evaluation of an adrenal lesion. Hypertension was observed in only one patient with hereditary syndrome and similarly hypertension was not observed in any pheochromocytoma patients who were diagnosed incidentally. Genetic testing is not available in our center; hence all patients who were diagnosed with MTC underwent further biochemical and cross-sectional imaging to rule out pheochromocytoma and primary hyperparathyroidism.

Prior to 2015, 24-hour urinary VMA was the screening test for pheochromocytoma being used at our center. All six patients had normal urinary VMA level but on histopathology were proven to be pheochromocytoma. These results are in accordance with low sensitivity of this test as reported by Lenders et al. in 2002 [24]. Plasma metanephrine and normetanephrine levels were checked in 19 patients and were raised in all of them. This adheres to international literature in which specificity and sensitivity of this test has been reported as 98 and 100% [25]. This study also proved that when compared between 24 hour urinary VMA and plasma metanephrines, the later provide the best test for excluding or confirming pheochromocytoma and should be the preferred test for diagnosis of the tumor among the two. Cross-sectional CT/MRI was able to correctly localize all tumors preoperatively, similar to other studies [19,22]. MIBG correctly identified the tumor in all cases, with MIBG sensitivity being 100% (n = 20); this is slightly higher than that reported by Berglund et al. and Shapiro study which was 88-98%, and may be related to large tumor sizes [26,27].

An open adrenalectomy was performed in majority of our patients (86.2%), most likely in view of large tumor size identified on preoperative imaging. Perioperative complications were present in about 50% of our patient which is consistent with literature but is slightly higher than that reported by Kinney et al. [28]. This relatively higher complication rate in our study is likely due to absence of alpha and beta blockade in some patients per operatively as has been stated in results.

Tumor size in our study (mean 8.3 cm) was larger than that recorded in other studies like Lo et al. and Zorgani et al., in both studies mean tumor size reported 6.4 cm [19,29]. This may be accounted for by late presentation or delayed referral for specialized care. Biochemical, structural and clinical cure was achieved in 96.4% of our patients. This cure rate is higher (70-92.4%) than that reported in other studies [2,19,23]. The reason for this higher cure rate can be as there was only one patient with malignant pheochromocytoma in our study and hence with metastatic disease, secondly follow-up period in our patients (mean follow-up 16.5 months) was less than that in other studies. Overall mortality rate in pheochromocytoma is low and various studies like Zorgani et al., Huddle, have reported it to be 5.9-7.4% [23,29]. Our study also showed a low mortality rate of 6.9%. The reason for low mortality is due to adequate alpha and beta blockade in perioperative period, in our study patients did experience higher perioperative complications rate but those were adequately managed and overall mortality was low and none from perioperative complications.

This study has several limitations. A retrospective study was performed but data was extracted using electronic hospital software and collaborated with patient visits to other clinics within the hospital. This would reduce recall any selection bias which traditionally makes retrospective studies weak, standardized biochemical testing was not performed for all pheochromocytoma patients, it was a single center study, and although pheochromocytoma is a rare diagnosis the sample size of patients was small. However, regardless of small number, this is a novel study as there is scarcity of data available regarding clinical presentation and outcome of pheochromocytoma in this part of subcontinent.

## Conclusions

This study highlights the importance of keeping high index of suspicion for pheochromocytoma at primary health care levels which would lead to early referrals. Furthermore, pheochromocytoma can have varying presentations and hypertension is not necessarily a predominant presentation. More studies are required specially from this part of subcontinent to further confirm the results of this study.
